# Genetic variation of temperature-regulated curd induction in cauliflower: elucidation of floral transition by genome-wide association mapping and gene expression analysis

**DOI:** 10.3389/fpls.2015.00720

**Published:** 2015-09-10

**Authors:** Claudia Matschegewski, Holger Zetzsche, Yaser Hasan, Lena Leibeguth, William Briggs, Frank Ordon, Ralf Uptmoor

**Affiliations:** ^1^Chair of Agronomy, Faculty of Agriculture and Environmental Science, University of RostockRostock, Germany; ^2^Institute of Resistance Research and Stress Tolerance, Julius-Kuehn InstituteQuedlinburg, Germany; ^3^Institute of Horticultural Production Systems, Leibniz Universität HannoverHannover, Germany; ^4^Syngenta Seeds B.V., EnkhuizenNetherlands

**Keywords:** genome-wide association study (GWAS), quantitative trait loci (QTL), transcriptional profiling, single nucleotide polymorphism (SNP), linkage disequilibrium (LD), vernalization, cauliflower, curd induction

## Abstract

Cauliflower (*Brassica oleracea* var. *botrytis*) is a vernalization-responsive crop. High ambient temperatures delay harvest time. The elucidation of the genetic regulation of floral transition is highly interesting for a precise harvest scheduling and to ensure stable market supply. This study aims at genetic dissection of temperature-dependent curd induction in cauliflower by genome-wide association studies and gene expression analysis. To assess temperature-dependent curd induction, two greenhouse trials under distinct temperature regimes were conducted on a diversity panel consisting of 111 cauliflower commercial parent lines, genotyped with 14,385 SNPs. Broad phenotypic variation and high heritability (0.93) were observed for temperature-related curd induction within the cauliflower population. GWA mapping identified a total of 18 QTL localized on chromosomes O1, O2, O3, O4, O6, O8, and O9 for curding time under two distinct temperature regimes. Among those, several QTL are localized within regions of promising candidate flowering genes. Inferring population structure and genetic relatedness among the diversity set assigned three main genetic clusters. Linkage disequilibrium (LD) patterns estimated global LD extent of *r^2^* = 0.06 and a maximum physical distance of 400 kb for genetic linkage. Transcriptional profiling of flowering genes *FLOWERING LOCUS C* (*BoFLC*) and *VERNALIZATION 2* (*BoVRN2*) was performed, showing increased expression levels of *BoVRN2* in genotypes with faster curding. However, functional relevance of *BoVRN2* and *BoFLC2* could not consistently be supported, which probably suggests to act facultative and/or might evidence for *BoVRN2*/*BoFLC*-independent mechanisms in temperature-regulated floral transition in cauliflower. Genetic insights in temperature-regulated curd induction can underpin genetically informed phenology models and benefit molecular breeding strategies toward the development of thermo-tolerant cultivars.

## Introduction

In cauliflower, curd development is regulated by temperature since vernalization is obligatory to promote the transition from the vegetative to the generative phase and thus, to induce the formation of the edible curd as marketable plant organ. Vernalization requirements can only be fulfilled after passing through a juvenile vegetative phase during which plants are insensitive to vernalization (Wurr et al., 1993). The length of the juvenile phase as well as vernalization rates and temperature optima considerably vary among ecotypes, while vernalization requirements have been reported to range from obligate (e.g., cool-temperate spring cultivars) to facultative (e.g., tropical cultivars; Friend, 1985). High ambient temperature can prolong the vegetative development phase by delayed vernalization and strongly impair harvest traits leading to high fluctuations in market supply and pricing. Moreover, exposure to excessive heat during the vernalization-sensitive phase often results in bracting (Fujime and Okuda, 1996; Grevsen et al., 2003), whereas low temperature can induce premature flower bud development that causes riciness (Grevsen et al., 2003), both accounting for highly reduced curd qualities. Due to the strong impact of temperature on curd development that defines economic value, evaluating temperature-regulated curd induction is of serious agronomic interest in cauliflower production. However, genetic mechanisms of floral transition are poorly understood and available phenology models intended to predict vernalization and harvest traits still hold inaccuracy. Thus, dissecting the genetic basis of flowering time is essential to predict and manage harvest traits. The identification of genomic markers associated with temperature response and flowering stability will accelerate marker-assisted breeding efforts toward the development of uniformly developing cultivars with predictable harvest traits adapted to a wide range of environments.

Flowering time is a complex trait that is regulated by an intricate signaling network of multiple genes that integrates endogenous and exogenous stimuli, such as vernalization and photoperiod, to induce flowering at the most favorable conditions (Boss et al., 2004; Amasino, 2005). The molecular mechanisms underlying floral transition in plants have been mainly elucidated in *Arabidopsis thaliana* (Mouradov et al., 2002; He and Amasino, 2005). However, the understanding of the genetic and epigenetic basis of floral transition still requires a sizable body of investigation. Benefiting from genetic analysis of mutants and natural variation in *Arabidopsis*, several flowering genes have been identified. General flowering pathways and main flowering time genes seem to be conserved in several agronomically important crops, among them the closely related *Brassica* species, including cauliflower (Lagercrantz et al., 1996; Schranz et al., 2006). Consequently, these findings enable the transfer of proposed flowering time models from *Arabidopsis* to important crop species. In the vernalization pathway, *FLOWERING LOCUS C* (*FLC*) and *VERNALIZATION 2* (*VRN2*) play a pivotal role in the regulation of the transition from vegetative to generative phase (Schmitz and Amasino, 2007). *FLC* encodes a MADS-box protein that prevents floral transition by directly repressing floral integrators, among them *FLOWERING TIME* (*FT*), *FLOWERING LOCUS D* (*FD*), and *SUPPRESSOR OF OVEREXPRESSION OF CONSTANS 1* (*SOC1*; Michaels and Amasino, 1999; Alexandre and Henning, 2008; Deng et al., 2011). In response to vernalization-inductive temperature, expression of *VERNALIZATION 2* (*VRN2*), together with *VERNALIZATION 1* (*VRN1*) and *VERNALIZATION INSENSITIVE 3* (*VIN3*) is induced, which mediates repression of *FLC* and thus releases flowering genes *FT, FD*, and *SOC1* from *FLC* suppression (He and Amasino, 2005; Jung and Müller, 2009). This in turn, results in subsequent activation of meristem identity genes, e.g., *LEAFY* (*LFY*) and *APETALA 2* (*AP2*) that promote floral induction (Sung and Amasino, 2004; Jung and Müller, 2009). Comparative analysis revealed several homologs of flowering time genes in *Brassica* crops with conserved regulatory function (Lagercrantz et al., 1996; Schranz et al., 2006). In *Brassica oleracea*, four FLC homologs have been identified, while only *BoFLC2* has been suggested to be of regulatory relevance in vernalization-induced flowering (Okazaki et al., 2007).

Numerous studies on floral transition in *Brassica* crops identified several quantitative trait loci (QTL) for flowering time that mapped to genomic regions displaying synteny to the region of *Arabidopsis* chromosome 5 harboring several flowering genes (Rae et al., 1999; Okazaki et al., 2007; Razi et al., 2008). In a doubled haploid (DH) population of *B. oleracea*, flowering time QTLs were detected on chromosomes O2, O3, O5 and O9, which are syntenic to *Arabidopsis* chromosome At5 (Bohuon et al., 1998). In a mapping population of a *B. oleracea* var. *italica* x *B. oleracea* var. *capitata* cross, a major QTL for flowering time was located on O2, where *BoFLC2* is mapped, suggesting its potential role in controlling floral transition (Okazaki et al., 2007). In contrast, other studies on flowering time variability in *B. oleracea*, observed no co-segregation of the *BoFLC* locus with flowering time and suggested *FLC*-independent pathways (Ridge et al., 2014). However, a *FLC*-independent vernalization pathway for flowering in *B. oleracea* has not been described yet.

Beyond QTL mapping, genome-wide association studies (GWAS) have advanced as a promising approach recently emerged in crop improvement to identify genes and distinct genetic variants controlling complex traits with respect to natural variation (Korte and Farlow, 2013). GWAS has been successful implemented in *A. thaliana* to elucidate the impact of natural variation on genetic variance of flowering time pathways (Atwell et al., 2010; Brachi et al., 2010) but also to study quantitative traits in other agronomically relevant crops, like rice, barley and maize (Buckler et al., 2009; Huang et al., 2012; Rode et al., 2012; Wang et al., 2012).

The present study aims at genetic dissection of temperature-related curd induction in cauliflower by combining genome-wide association mapping and gene expression analysis conducted on a cauliflower diversity set, consisting of 111 accessions. In particular, the main objectives of the study are (i) assessment of phenotypic variation of curd induction in dependence on temperature, (ii) detection of genomic markers significantly associated with flowering time by GWAS, (iii) identification of promising QTL regions and putative candidate genes, (iv) evaluation of genetic diversity and linkage disequilibrium (LD) patterns among the cauliflower diversity set, and (v) transcriptional analysis of flowering time genes to examine its functional relevance during vernalization and curd induction in cauliflower. Results will give insights in genetic regulation of temperature-related curd induction in cauliflower and further contribute to the elucidation of molecular pathways underlying floral transition. Especially, as interest in the development of genetically informed models describing flowering is recently raised (Wilczek et al., 2009), the high potential of combining GWAS and linkage mapping, additionally underpinned with gene expression data, is demonstrated as a promising step forward toward the understanding of genetic traits that determine natural variation and inform plant breeding.

## Materials and Methods

### Plant Material and Genotyping

For the experiments, a diversity set consisting of 111 *B. oleracea* var. *botrytis* commercial parent lines was used. The diversity panel included lines from the temperate zone (*n* = 99), comprising early (*n* = 5), medium (*n* = 63) and medium-long (*n* = 31) time to harvest varieties and accessions from the subtropical (*n* = 3) and tropical (*n* = 9) zones. These materials display variation in temperature-related curd induction and harvest time reliability. For genotyping, DNA was extracted from freeze-dried leaf tissue and hybridized to an Illumina Infinium iSelect (*B. oleracea* 20k) array according to the manufacturer’s protocol. Array hybridization resulted in 14,385 polymorphic SNP markers used for genotyping, covering the whole cauliflower genome and being equally distributed among all linkage groups (O1–O9).

### Greenhouse Trials and Phenotyping

Two independent greenhouse trials under distinct temperature regimes were carried out on the whole cauliflower diversity set, conducted under: (i) semi-controlled conditions, where plants were cultivated in the greenhouse under natural light and photoperiod conditions with average daily minimum and maximum temperature of 15.0 ± 4.0°C and 30.7 ± 6.3°C, respectively (GH-T1) and (ii) controlled temperature conditions applying high ambient stress temperature, consistently not lower than 22.5°C with a photoperiod of 12/12 h day/night (GH-T2). In GH-T1, plants were sown on 6th May 2013, in GH-T2 on 10th October 2013, in trays filled with modular tray substrate (Klasmann-Deilmann GmbH; Geeste, Germany) and cultivated in the greenhouse. After four weeks, plants were potted in 1.7 l pots filled with clay substrate (Klasmann-Deilmann GmbH; Geeste, Germany). Plants were fertigated frequently including application of Universol Orange fertilizer (16% total N, 5% P_2_O_5_, 25% K_2_O; Everris, The Netherlands) with increasing concentrations from 5 to 26 g x l^-1^. Plants were treated with SpinTor (Dow AgroSciences; Indianapolis, IN, USA) and KARATE ZEON (Syngenta Seeds; Basel, Switzerland) against cabbage root fly and cabbage whitefly. Temperature was recorded hourly with a Tinytag Ultra 2 data logger (Gemini Data Loggers, Ltd; Chichester, UK). Experimental trials were performed with four plants per accession in a randomized complete block design with two replications. Plants were phenotyped frequently, at least three times a week, until visible curd induction occurred. The onset of visible curd initiation was recorded by the number of days after sowing (DAS). For accessions which did not induce a curd at the end of the experiment, the termination date of the experiments (105 DAS for GH-T1 and 112 DAS for GH-T2) was assigned, since data elimination of non-curding genotypes under high temperatures might limit GWAS power in terms of detecting key loci. Accessions with less than three plants per line where excluded from data analysis.

### Statistical Analysis of Phenotype Data

Statistical analysis of phenotypic data was performed by Kruskal-Wallis test with statistical software SPSS Statistics 22. Statistical significance was assumed at *P* < 0.05. One-way analysis of variance (ANOVA) was carried out with statistical software R^1^ and variance components were calculated as functions of the mean squares estimated from the ANOVA. Broad-sense heritability (*h*^2^) was calculated according to Hill et al. (1998) as follows:

$$h^{2}\,=\;\frac{\sigma_{G}^{2}}{\sigma_{G}^{2}+\sigma_{GxE}^{2}/e+\sigma_{\varepsilon }^{2}/re}$$

where σ^2^*_G_* is the genetic variance, σ^2^*_GxE_* is the genotype x environment interaction variance, σ^2^*_ε_* is the residual variance and *e* and *r* are the numbers of environments and replications per environment, respectively.

### Population Structure and Kinship

The population structure of the diversity panel was estimated with STRUCTURE software 2.3.4 (Pritchard et al., 2000a) applying a model-based Bayesian approach. This approach also allows for correction in terms of population substructure and genetic relatedness in order to avoid false-positive associations in association mapping studies (Pritchard et al., 2000b). STRUCTURE analysis was performed for *K* = 1–10 clusters with five replications for each *K*-value comprising 100,000 burn-in period iterations followed by 100,000 Markov Chain Monte Carlo iterations while using a population admixture ancestry model. The determination of the most probable number of subpopulations (*K*) within the diversity panel was based on the calculation of Δ*K* according to Evanno et al. (2005). Genotypes were then assigned to the respective subpopulation showing the highest estimated membership coefficient (*Q)*. Admixture was assumed if the *Q*-value for the probability to belong to one subgroup was < 100%. The cluster analysis was performed with the neighbor-joining algorithm (Saitou and Nei, 1987) implemented in TASSEL software 4.0 (Bradbury et al., 2007).

### Association Mapping and Linkage Disequilibrium

Genome wide association (GWA) analysis was performed with TASSEL 4.0 (Bradbury et al., 2007) by applying a mixed linear model (MLM), accounting for population structure previously estimated with STRUCTURE as a fixed effect matrix (*Q*-matrix) and kinship (*K*-matrix), calculated with TASSEL, as a random effect matrix accounting for genetic relatedness to reduce false-positive results. For performing association analysis, SNPs were filtered according to minor allele frequency (MAF) and call rate, excluding those with a MAF < 0.05 and a minimum call rate < 0.9, finally resulting in 4,758 out of 14,385 SNPs considered in GWA mapping. Significant marker association with the phenotypic trait was assumed for markers exhibiting a *P*-value < 0.01. Candidate gene search was performed by using the publicly available *B. oleracea* var. *capitata* genome database (Oil Crops Research Institute^2^; Yu et al., 2013) and the *Arabidopsis Information Resource (TAIR*^3^). LD as represented by squared allele frequency correlations (*r^2^*), between each pair of marker loci (Pritchard and Przeworski, 2001) was estimated with TASSEL 4.0 (Bradbury et al., 2007). Only alleles with a frequency >0.05 were considered for the calculation of LD because *r^2^* exhibits large variances if rare alleles are considered (Wen et al., 2009). Pairs of loci were considered to be in significant LD if *r*^2^ values were significant at *P* < 0.05. The LD was estimated separately for loci on the same chromosome (intrachromosomal marker pairs) and for unlinked loci (interchromosomal marker pairs). LD decay was estimated by using second-degree locally weighted polynomial regression (LOESS; Cleveland, 1979), obtained with the statistical software R^1^, of intrachromosomal *r^2^* values plotted against physical distance. LD between interchromosomal markers (unlinked) was estimated to determine critical *r^2^* value, as a population-specific threshold above which LD is assumed to be caused by genetic linkage (Breseghello and Sorrells, 2006). Critical *r^2^* results from the 95% percentile of the distribution of root-transformed *r^2^* estimates. The determination of the physical distance at which the LOESS curve intercepts the critical *r^2^* defines the LD decay rate. In addition, SNPs significantly associated with the trait obtained from GWAS were analyzed for local LD patterns. Physically neighbored SNPs exhibiting genetic linkage were clustered in order to correct for total number of significant marker-trait associations. The LD plot was constructed with Haploview software 4.2 (Barrett et al., 2005).

### RNA Isolation, Reverse Transcription, and qRT-PCR Analysis

For qRT-PCR analysis, fresh leaf samples were collected at seven time points, starting after germination until the onset of visible curd induction. In detail, sampling dates were 22, 36, 43, 57, 64, 71, 78 DAS. At each time point, samples of ∼100 mg of fresh tissue of the lateral tip of the second fully developed leaf were harvested, immediately frozen in liquid nitrogen and stored at -80°C. Standardized sampling conditions were applied in order to avoid circadian or diurnal variation in gene expression.

Total RNA was isolated from leaf tissue using the NucleoSpin^®^ RNA plant isolation kit (Macherey-Nagel, Düren, Germany) according to the manufacturer’s instruction. In addition to the standard protocol, a second DNA digestion step with DNase I (Thermo Scientific, Braunschweig, Germany) was added to completely remove genomic DNA. Absence of genomic DNA contamination was confirmed by PCR analysis using the isolated RNA as a template as suggested by Udvardi et al. (2008). RNA integrity was assessed by visualizing *28S* and *18S* rRNA bands under UV light in a denaturing agarose gel stained with ethidium bromide. RNA concentration was measured using a Nanodrop ND-1000 spectrophotometer^4^. Complementary DNA (cDNA) was synthesized from 2.5 μg of total leaf RNA using the RevertAid First Strand cDNA Synthesis Kit (Thermo Scientific, Braunschweig, Germany) following the manufacturer’s protocol. qRT-PCR of temperature-related flowering time genes *BoFLC2* and *BoVRN2* was performed using the CFX96 Real Time PCR Detection System and SYBR fluorescence (Bio-Rad Laboratories, München, Germany) for detection. To normalize gene expression, the constitutively expressed *18S* was selected as internal reference (Pfaﬄ, 2001). Gene-specific primers (Supplementary Table S1) were designed using Primer 3 software^5^ based on the published genome sequence of *B. oleracea* var. *capitata* (Oil Crops Research Institute^2^; Yu et al., 2013) and the *B. oleracea* var. *italica* Short Read Archive^6^ (SRA accession SRX032205, BioSample ID SAMN001138238). Primers were synthesized by Biolegio (Nijmegen, The Netherlands). To exclude cross-binding to other targets than to the genes of interest, *in silico* designed primer sequences were again subjected to BLAST analysis in the *B. oleracea* var. *capitata* genome database and cross-validated with the *B. oleracea* var. *italica* Short Read Archive. Additionally, high resolution melting curve analysis was performed after each qPCR assay to verify that qPCR assays have amplified single, specific products and thus to ensure gene-specificity of the primers. Melt curve was generated within the range of 65–95°C in increments of 0.5°C per 0.05 s. Gel electrophoresis further was conducted to reconfirm that a single amplicon was amplified.

The qRT-PCR reaction mixtures were prepared according to the protocol of Maxima^TM^ SYBR Green qPCR Master Mix kit (Thermo Scientific, Braunschweig, Germany). Three technical replicates of qPCR assay were performed for each gene, while comprising three biological replicates, respectively. The data were analyzed using the Bio-Rad CFX Manager software (Bio-Rad Laboratories, München, Germany). Relative gene expression was calculated according to the ΔΔC_T_ method. The average C_T_ of the reference control gene was used to normalize gene expression data; C_T_ values of candidate genes were subtracted by C_T_ of the reference gene (ΔC_T_). To calculate ΔΔC_T_, ΔC_T_ of interest was subtracted by the ΔC_T_ of control. These results were transformed to log2 scale to obtain the fold-change values.

## Results

### Phenotypic Variation in Temperature-Related Curd Induction in Cauliflower

**FIGURE 1:**
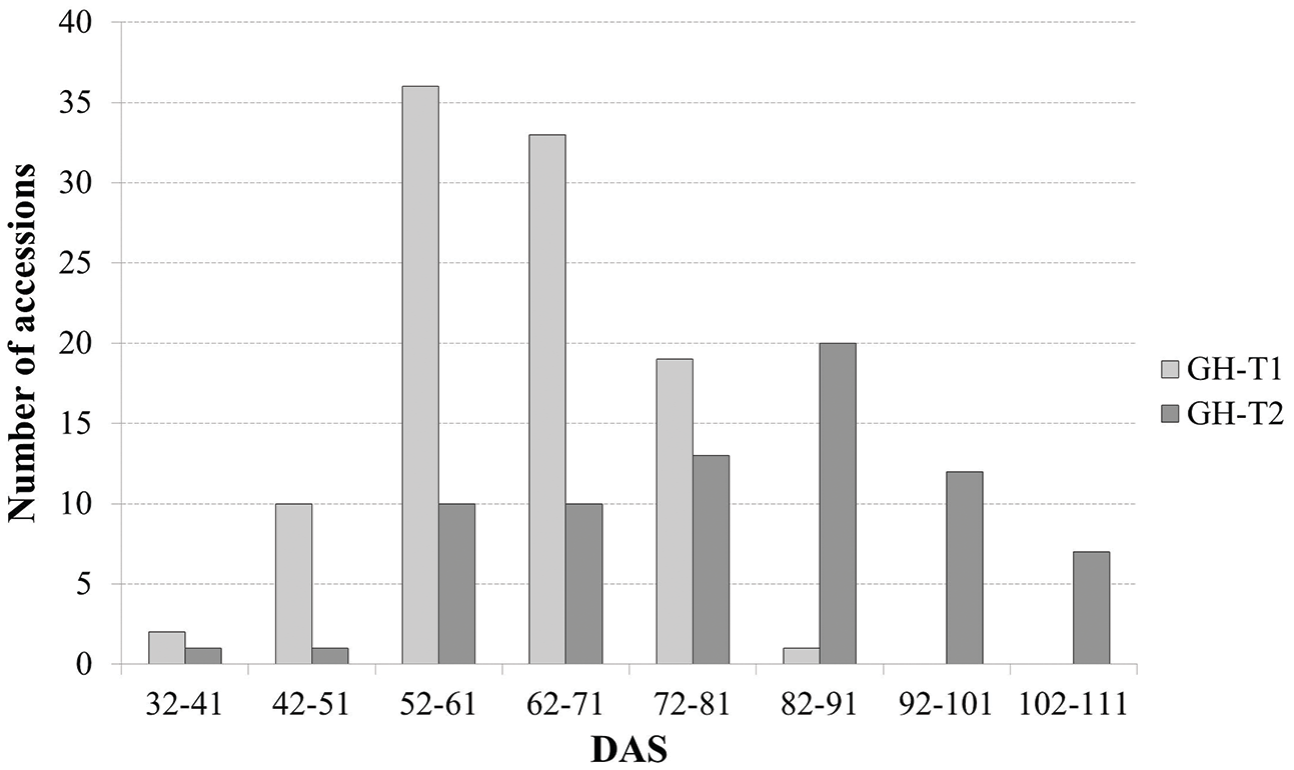
**Frequency of days after sowing (DAS) to visible curd induction among the cauliflower diversity set under semi-controlled temperature conditions (GH-T1) and under high ambient temperatures of 22.5°C (GH-T2)**.

Phenotypic data is summarized in **Table 1**. Lines which did not induce a curd at the end of the experiment or lines with less than three plants were excluded, resulting in 107 lines from greenhouse trial GH-T1 and 88 lines from GH-T2 remained for final data analysis. With regard to distinct temperature regimes, mean time to curd induction among all accessions of the diversity set was lower in GH-T1 with 61.9 ± 9.7 DAS compared to 79.7 ± 15.5 DAS in GH-T2, performed under high ambient temperature of constantly not lower than 22.5°C. Minimum and maximum time to curd induction ranged from 32.0 to 84.0 DAS in GH-T1 compared to 40.3 to 106.3 DAS in GH-T2. The latter generally showed higher phenotypic variation in time to curd induction (**Figure 1**). Broad-sense heritability for time to curd induction was high as estimated with *h*^2^ = 0.93.

**Table 1:** Summary statistics of variation of curd induction as measured in days after sowing (DAS) under natural temperature regime (GH-T1) and high ambient temperature (GH-T2) in the greenhouse including coefficients of variance and heritability (*^∗∗∗^P* < 0.001).

	GH-T1	GH-T2
**Curd induction (DAS)**		
Mean ± SD	61.9 ± 9.7	079.7 ± 15.5
Minimum ± SD	32.0 ± 0.0	040.3 ± 2.80
Maximum ± SD	84.0 ± 0.0	106.3 ± 2.90
**Variance components**		
σ^2^_*c*_	879.4^∗∗∗^	
σ^2^_*GxE*_	62.6^∗∗∗^	
σ^2^_𝜀_	126.3	
*h*^2^	0.93	

*σ^2^_c_ Genotypic variance*

*σ^2^_GxE_ Genotype x environment variance*

*σ^2^_𝜀_ Error variance*

*h^2^ Heritability*

*SD Standard deviation*

**FIGURE 2:**
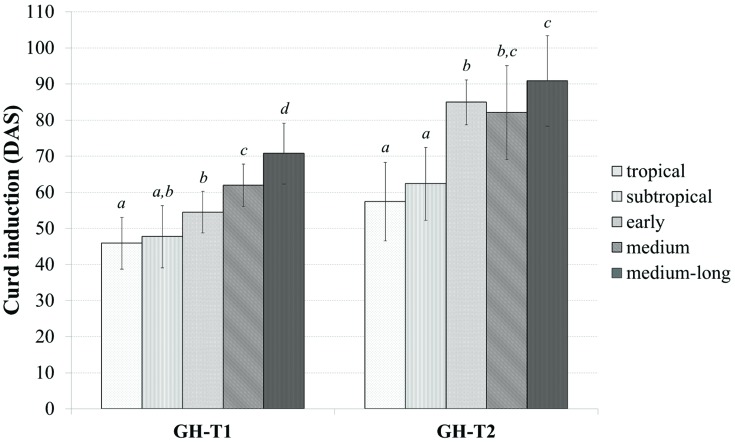
**Phenotypic variation of curd induction (measured in DAS) among the cauliflower diversity set according to the classification into distinct groups consisting of early, medium, medium-long, subtropical and tropical accessions under a natural temperature regime (GH-T1) and high ambient temperatures of 22.5°C (GH-T2; mean ± SD, *P* < 0.05, Kruskal–Wallis test; similar letters indicate no significant statistical differences)**.

Classifying the plant material into tropical, subtropical and early, medium, and medium-long harvest time lines from the temperate zone, the mean time to curd induction was higher under the high ambient temperatures of GH-T2 for all groups (**Figure 2**). In particular, mean time to curd induction was shortest for those accessions selected in the tropical and subtropical climate with 45.9 ± 7.2 and 47.7 ± 8.6 DAS for GH-T1 and 57.4 ± 10.9 and 62.3 ± 10.1 for GH-T2, respectively. Mean time to curd induction of lines from the temperate zone was generally higher.

### Population Structure and Genetic Relatedness

**FIGURE 3:**
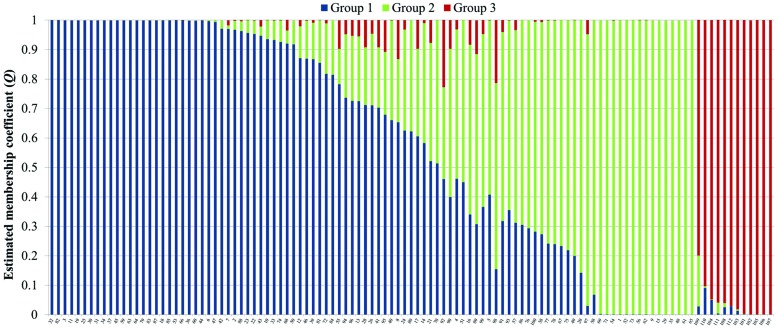
**Bayesian analysis of population structure in the cauliflower diversity panel assigning the accessions to three subpopulations.** Each genotype is represented by a vertical bar, which is partitioned into *K* colored segments that represent the individual’s estimated membership coefficient (*Q*) to the *K* clusters (STRUCTURE 2.3.4).

**FIGURE 4:**
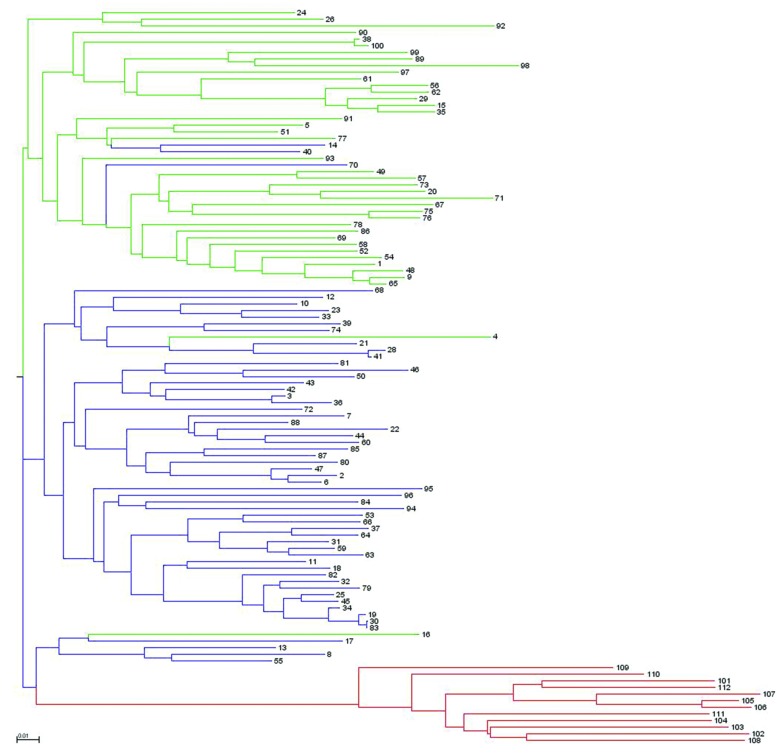
**Neighbor-joining dendrogram showing genetic relatedness among the 111 cauliflower accessions of the diversity panel based on 4,758 SNP markers.** Accessions are color-coded according to the populations’ substructure assignment to cluster G1, G2, and G3 based on STRUTURE results (see **Figure 3**).

Analysis of population structure by admixture model-based simulations, determines *K* = 3 as the most likely number of subpopulations calculated according to Evanno et al. (2005; Supplementary Figure S1). STRUCTURE analysis assigned the accessions of the diversity set to three main subpopulations, denoted as G1, G2 and G3 comprising 61, 38 and 12 accessions, respectively (**Figure 3**). Thereby, a distinct separation of accessions from the temperate zone (clustering in G1 and G2) from those of the tropical/subtropical zone (G3) could be observed. In detail, G1 (*n* = 61) was composed of early (*n* = 5), medium (*n* = 27) and medium-long (*n* = 29) term varieties from the temperate zone, whereas G2 (*n* = 38) mainly comprises medium varieties from the temperate zone (*n* = 36) and two medium-long term accessions. G3 (*n* = 12) includes only tropical and subtropical lines. A total of 42 accessions (37.8%) showed a clear relationship each with one single cluster based on their inferred ancestry value of 100%, while the remaining 69 accessions (62.2%) were categorized as admixtures. Neighbor-joining analysis based on Nei’s genetic distance was performed to display the genetic relationships among the 111 cauliflower accessions (**Figure 4**). Neighbor-joining analysis showed similar classification into three subpopulations widely in consistency with STRUCTURE analysis. The neighbor-joining tree consists of three main branches. Two of the main branches nearly exactly display the subpopulations G1 and G2 of the STRUCTURE analysis. The third main cluster consists of two distinct branches, one of them comprises all of the G3 genotypes, the other one consists of five genotypes, belonging to G1 and G2. When looking at the estimated membership coefficients (*Q*) for those genotypes, it becomes obvious, that they display a relatively high degree of admixture.

### Whole Genome Patterns of Linkage Disequilibrium (LD)

**FIGURE 5:**
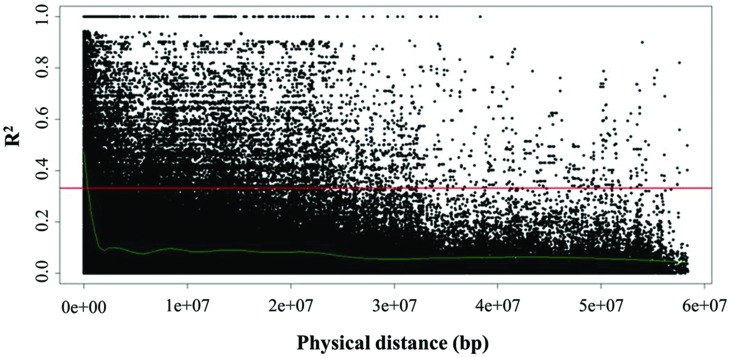
**Linkage disequilibrium (LD) represented as *r^2^* of all intrachromosomal marker pairs plotted against physical distance among all linkage groups (O1–O9).** The horizontal line (*red*) marks critical *r^2^* (*r^2^* = 0.33) as threshold beyond which LD is assumed to result from genetic linkage. The intersection point with the non-linear regression LOESS curve (*green*), which determines LD decay, marks the physical distance as threshold for the maximum distance between genetically linked markers.

Global extent of LD decay as well as local LD for each of the linkage groups O1–O9 was inferred by pairwise comparison of LD estimates (*r^2^*) of interchromosomal (unlinked) and intrachromosomal (linked) marker pairs (**Figure 5**). Average global LD was estimated at *r^2^* = 0.06 with 20.71% interchromosomal marker pairs in significant LD (*P* < 0.05). Average intrachromosomal LD for significant marker pairs values at *r^2^* = 0.23 (*P* < 0.05), while ranging from 0.18 to 0.28 among the linkage groups. LD estimates were significant for 38.40% intrachromosomal marker pairs (*P* < 0.05), with 2.87% marker pairs that were completely linked (*r^2^* = 1). However, *r^2^* decreased as the physical distance between loci increased, indicating that the probability of LD was low between distant marker pairs. Estimation of LD between interchromosomal markers (unlinked) was inferred to determine critical *r^2^* value, as a population-specific threshold above which LD is assumed to be caused by genetic linkage (Breseghello and Sorrells, 2006). Critical *r^2^* was estimated at *r^2^* = 0.33. The intersection point of critical *r^2^* with the non-linear regression LOESS curve was at ∼400 kb, describing LD decay, i.e., maximum physical marker distance for genetically linked markers. While considering critical LD at *r^2^* = 0.33, 20.5% marker pairs were in LD since their *r^2^* values were higher than 0.33.

### GWA Mapping on Temperature-Related Floral Induction

**FIGURE 6:**
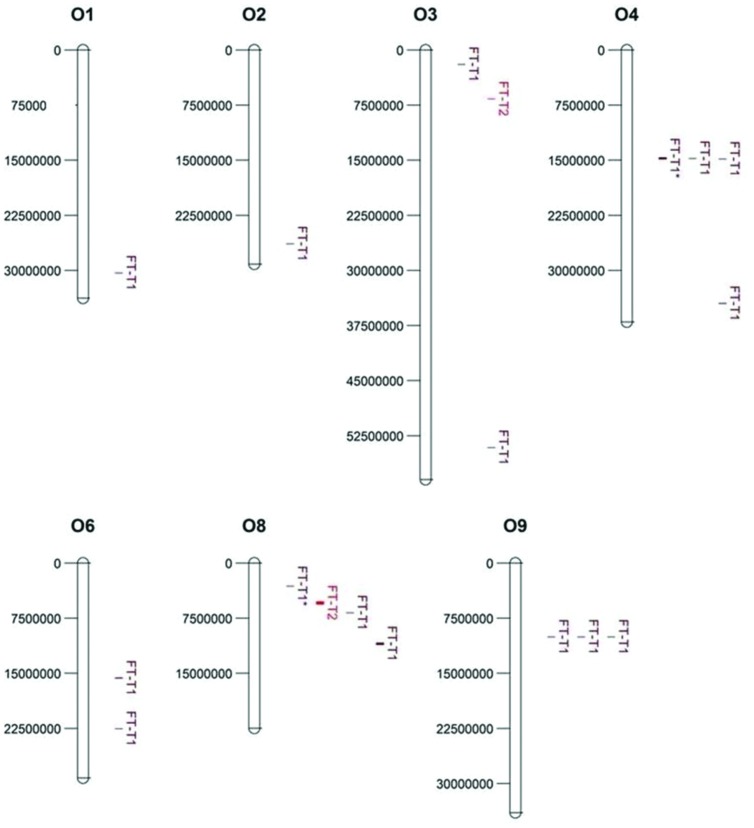
**Physical map and chromosomal position of significant QTL (*P* < 0.01) associated with temperature-related curd induction in cauliflower under natural temperature regime (GH-T1) and under high ambient temperatures of 22.5°C (GH-T2), denoted as FT-T1 (*dark red*) and FT-T2 (*red*), respectively.** Significant QTL are located on chromosomes O1, O2, O3, O4, O6, O8, and O9 (*left*: physical position in bp). Two significant FT-T1 QTL on O4 and O8, marked with an asterisk (FT-T1^∗^), were genetically linked (*r^2^* = 0.7).

**FIGURE 7:**
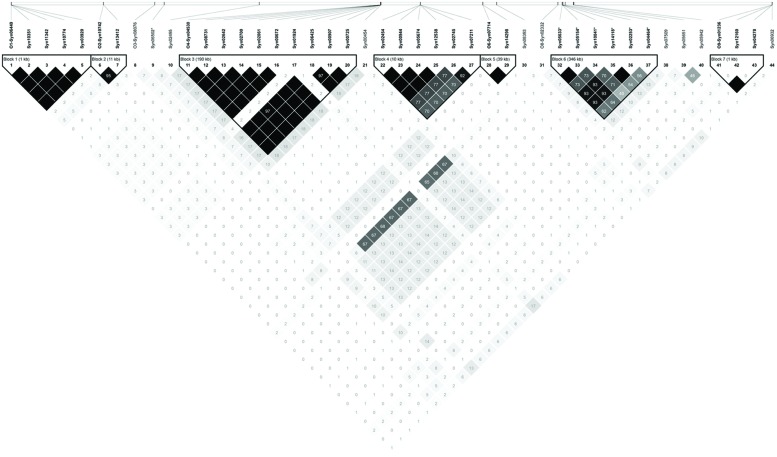
**Linkage disequilibrium plot showing LD patterns among significant SNPs for temperature-related curd induction.** The LD between the SNPs is measured as *r^2^* and shown (×100) in the square at the intersection of the diagonals from each SNP. *r^2^* = 0 is shown in white, 0 < *r^2^* < 1 is shown in gray and *r^2^* = 1 is shown in black. The analysis track at the top shows the SNPs according to their chromosomal location; while separating linkage groups by O1-, O2-, O3-, O4-, O6-, O8-, and O9- prefix, respectively. Seven haplotype blocks (outlined in bold black line) indicate markers that are in high LD.

Association mapping revealed a total of 44 significant marker-trait associations (*P* < 0.01) for curd induction under both temperature regimes. Chromosomal position, *P*-level of significance and allele effects for all detected markers significantly associated with temperature-related curd induction are summarized in Supplementary Table S2. For GH-T1, a total number of 37 significant SNPs were detected (*P* < 0.01) on chromosomes O1 (*n* = 5), O2 (*n* = 2), O3 (*n* = 2), O4 (*n* = 17), O6 (*n* = 3), O8 (*n* = 4), and O9 (*n* = 4). For GH-T2, 7 significant markers on O3 (*n* = 1) and O8 (*n* = 6) associated with days to curd induction were detected. Genetic linkage between physically neighbored SNPs was assumed at *r^2^* = 0.33 (critical *r^2^* for genetic linkage, based on LD estimation in the cauliflower diversity set). By taking genetic linkage between intrachromosomal markers into account, it finally resulted in an adjusted number of 16 significant QTL regions for curd induction in GH-T1 (*P* < 0.01), localized on chromosomes O1 (*n* = 1), O2 (*n* = 1), O3 (*n* = 2), O4 (*n* = 4), O6 (*n* = 2), O8 (*n* = 3), and O9 (*n* = 3), and two QTL one each on O3 and O8 for GH-T2 (**Figure 6**). Among them, QTL positions on O3 and O8 in GH-T1 were not similar or overlapping with those detected in GH-T2. Local LD analysis for all detected significantly associated markers showed that suites of markers on O1, O2, O4, O6, O8, and O9 are in high intrachromosomal LD and could be assigned to a total of seven haplotype blocks (**Figure 7**) spanning marker intervals from 1 to 346 kb. In detail, haplotype blocks could be assigned to markers on O1 (*n* = 1; 1 kb), O2 (*n* = 1; 11 kb), O4 (*n* = 2; 190 and 10 kb), O6 (*n* = 1; 39 kb), O8 (*n* = 1; 346 kb), O9 (*n* = 1; 1 kb). In addition, LD analysis indicates genetic linkage among two significant QTL for GH-T1 detected on chromosomes O4 and O8 with *r^2^* = 0.7. In contrast, detected QTL on O3 and O8 in GH-T2 were not in LD and represent two individual QTL. A summary of the most promising QTL regions and putative candidate genes is shown in **Table 2**. A total of 9 QTL, detected in GH-T1 and GH-T2, could be found in genomic regions close to candidate genes involved in flowering regulation and vernalization response, located on chromosomes O1, O2, O3, O4, and O8. Among them, QTL for GH-T1 were found to be co-localized with, e.g., *BoFLC2* on O3, *BoVRN1* on O1 and *BoFLD* on O8. The QTL for GH-T2 on chromosome O3 was found in the genomic region close to *BoVIN3*.

**Table 2:** Summary of the most promising QTL and putative candidate genes involved in the regulation of flowering and vernalization response, located within detected QTL regions.

Chr.	QTL	Position (Mb)	Bolbase#	Start position (Mb)	Ortholog *Arabidopsis thaliana*	Gene name
1	FT-T1	30.35	Bol030990	30.98	AT3G18990	*VRN1, VERNALIZATION 1*
2	FT-T1	26.37–26.38	Bol016519	24.29	AT4G02560	*LD, LUMINIDEPENDENS*
3	FT-T1	1.96	Bol008758	1.89	AT5G10140	*FLC, FLOWERING LOCUS C*
			Bol034237	2.25	AT5G13480	*FY*
	FT-T2	6.66	Bol026036	5.91	AT5G57380	*VIN3, VERNALIZATION INSENSITIVE 3*
	FT-T1	54.11	Bol017452	54.09	AT4G31610	*REM1/-34, REPRODUCTIVE MERISTEM 1/-34*
			Bol013020	54.96	AT4G29830	*VIP3, VERNALIZATION INDEPENDENCE 3*
4	FT-T1	14.65–14.85	Bol038616	13.54	AT5G17690	*TFL2, TERMINAL FLOWER 2*
			Bol008371	16.07	AT2G28550	*RAP2.7, RELATED TO AP2.7*
	FT-T1	34.49–34.5	Bol014112	34.24	AT5G16320	*FRL1, FRIGIDA LIKE 1*
			Bol037895	35.54	AT2G33810	*SPL3, SQUAMOSA PROMOTER BINDING PROTEIN-LIKE 3*
8	FT-T1	3.14	Bol011554	2.47	AT3G10390	*FLD, FLOWERING LOCUS D*
	FT-T1	10.88–11.10	Bol027103	12.13	AT1G30970	*SUF4, SUPPRESSOR OF FRIGIDA 4*

### Transcriptional Profiling of Flowering Time Genes

**FIGURE 8:**
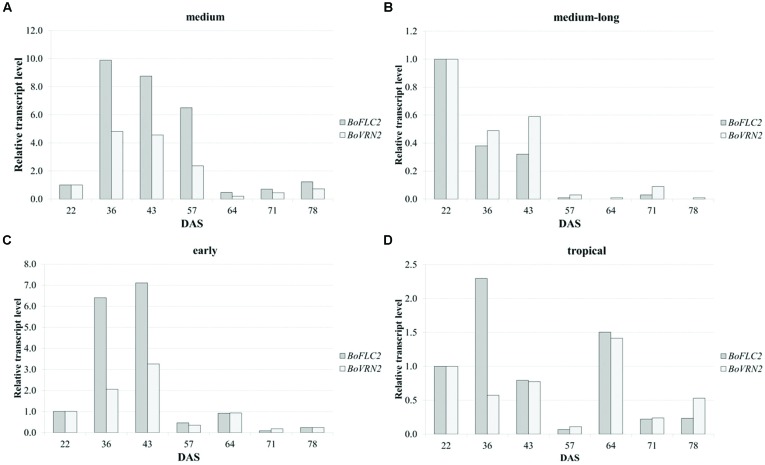
**Time-dependent relative changes in transcript levels of *BoFLC2* and *BoVRN2* during curd induction in medium (A), medium-long (B), early (C) and cauliflower accession from the temperate zone and the tropics (D).** Values represent mean relative changes in gene expression normalized to the control time point (22 DAS).

Quantitative real time PCR was carried out to analyze relative expression patterns of flowering time genes *FLOWERING LOCUS C 2* (*BoFLC2*) and *VERNALIZATION 2* (*BoVRN2*) and validate its functional relevance in temperature-related curd induction in cauliflower. Transcriptional profiling was conducted in four genotypes (*A–D*) comprising medium (*A*), medium-long (*B*) and early (*C*) variety from temperate zone and tropical climate (*D*) with diverging flowering phenotype with mean time to curd induction of 59.3 ± 4.0, 104.5 ± 3.5, 78.5 ± 5.9, 67.5 ± 5.4 DAS under high ambient temperature regime (GH-T2), respectively. These genotypes include both parental lines of the DH population on which previous QTL study has been conducted (unpublished data), referred to as genotype *A* (reliable parent) and genotype *B* (unreliable parent). Relative gene expression was analyzed in regard to temporal variation under high ambient temperature (GH-T2) at seven different time points ranging from 22, 36, 43, 57, 64, 71, 78 DAS (**Figure 8**). Genotype *A* (reliable parent) with the shortest time to curd induction under high ambient temperature (GH-T2) exhibits increased expression levels of both, *BoFLC2* and *BoVRN2* until the onset of visible curd induction (59.3 DAS), while *BoFLC2* transcript level was 9.88- to 6.5-fold higher in comparison to the control time point (22 DAS). In genotype *C* (early cultivar), similar expression patterns of *BoFLC2* and *BoVRN2*, possessing higher transcript levels until the onset of curd induction, were observed. After visible curd induction has occurred, *BoFLC2* and *BoVRN2* expression in genotype *A* and *C* decreased to a level similar to those of the control time point. In contrast, in genotype *B* (unreliable parent with the longest time to curd induction under high ambient temperature), relative gene expression of *BoFLC2* and *BoVRN2* was consistently reduced, while showing highly diminished values ranging between 0.01 and 0.59-fold expression compared to the control time point. In genotype *D* no general expression trends among *BoFLC2* and *BoVRN2* could be demonstrated.

## Discussion

Phenotypic analysis on curd induction under distinct temperature regimes in controlled greenhouse experiments displayed considerable variation in curd induction among the cauliflower diversity set. Mean time to visible curd induction was shorter under natural temperature regime (GH-T1) than under high ambient stress temperature (GH-T2). Similarly, time range for curding was smaller in GH-T1 when compared to GH-T2, consequently showing higher phenotypic variation and delayed curd induction under high ambient temperatures, which is consistent with available vernalization models, generally describing delayed vernalization with higher temperature (Wiebe, 1972a,b; Krug et al., 2002). Delay in curd induction under high ambient temperature (GH-T2) was lowest for varieties from tropical and subtropical climate, which indicates more tolerance to high temperature and/or less obligate vernalization requirements in contrast to those varieties originated from the temperate zone. The difference might be due to adaptation to distinct geographic habitats following selective breeding. A generally observed relation between the onset of curd induction and classification into groups, i.e., earlier curd induction for tropical and subtropical lines under high temperature than for varieties from the temperate zone, is in accordance to published phenology models describing cultivar-specific vernalization rates in dependence to temperature, while proposing a more broad range of vernalization-inductive temperature as well as a higher maximum vernalization rate at temperature optima for varieties derived from warmer climates than for those from the temperate zone (Wiebe, 1972a,b; Krug et al., 2002). High heritability (0.93) was estimated for temperature-related curd induction in the cauliflower diversity set, which is consistent with generally high heritability estimates for flowering in several crops, such as 0.7–0.9 in rapeseed (Long et al., 2007; Chaghakaboodi et al., 2012), 0.9 in rice (Seyoum et al., 2012; Ogunbayo et al., 2014) and 0.9 in barley (Maurer et al., 2015).

The observed variation in curd induction among the cauliflower diversity set regarding prior classification according to distinct geographical habitats corresponds well with the population structure estimated with STRUCTURE analysis. Tropical and subtropical accessions could be assigned to one cluster that clearly distinguished from accessions from the temperate zone, which clustered in two distinct groups. These results reconfirm that variation in flowering might be primarily based on genetic differences arising from artificial selection in different geographical areas or distinct environmental niches. The neighbor-joining dendrogram confirmed the STRUCTURE results by revealing similar clusters. Additionally, neighbor-joining analysis showed high genetic distance for tropical/subtropical accessions to accessions from the temperate zone, presenting less genetic relatedness between those accessions that might explain the high phenotypic variation in curd induction among those groups. For accessions from the temperate zone, a closer genetic relationship was observed, although two subgroups could be classified. However, a relatively large number of accessions were categorized as admixtures, with different proportion of mixed ancestry among the cauliflower population.

To dissect genetic variation in temperature-related curd induction, GWAS was conducted on the cauliflower diversity set. GWA analysis identified 16 QTL for temperature-related curding under natural temperature regime (GH-T1), whereby, under high ambient temperatures (GH-T2), only two loci significantly associated with the trait could be detected. In particular, genomic loci conferring temperature-regulated floral transition in cauliflower were mapped on *B. oleracea* chromosomes O1, O2, O3, O4, O6, O8, and O9 under temperature regime GH-T1 and 2 QTL one each on O3 and O8 under high ambient temperature (GH-T2). Thereby, QTL position on chromosomes O3 and O8 was not consistent among both temperature regimes suggesting that the QTL, solely expressed in GH-T2, might be specific for floral transition in terms of high ambient stress temperature. The results show, that under natural temperature conditions (GH-T1), curding and floral induction is controlled by several individual QTL, all of which might contribute with variable allelic effect. That is consistent with the concept that flowering is a multigenic trait, which is controlled by multiple QTL often with small genetic effects (Ferreira et al., 1995; Long et al., 2007; Zou et al., 2012; Raman et al., 2013). Previous QTL studies carried out on flowering time in *B. oleracea* give evidence for all of the detected QTL positions in this study. QTL analysis on a segregating population of F1-derived DH lines of *B. oleracea* var. *alboglabra* x *B. oleracea* var. *italica* identified QTL for temperature-regulated flowering on chromosomes O2, O3, O4, O5, O6, and O9 (Uptmoor et al., 2008). In a cross of cauliflower and Brussels sprout, QTL for flowering were detected on chromosomes O7 and O8, the latter being reported to account for maintenance of vernalization (Sebastian et al., 2002). Axelsson et al. (2001) mapped *FLC* homologs on O3 and O9 and identified flowering time QTL near this candidate gene. These results are consistent with the present study, representing, that many flowering time genes or temperature-associated genes were localized within significant marker intervals, suggesting that these genes may account for genetic variation in floral induction of cauliflower. However, these genes were localized within marker intervals of a few (1 kb) to several bp (346 kb). Consequently, high-resolution mapping of the identified QTL regions should be applied to reveal candidate genes putatively accounting for flowering time variation. But, as candidate gene search revealed, evidence is given for several flowering time genes, among them *FLC* and *VRN* as putative candidates to be involved in the regulation of floral transition and vernalization response in cauliflower, since some of the detected QTL could be found in genomic regions harboring those flowering genes.

The extent of LD, describing the non-random association of alleles between two loci in a population (Flint-Garcia et al., 2003) is affected mainly by factors such as genetic drift, population admixture, and selection as well as mating system, population size (Flint-Garcia et al., 2003; Gaut and Long, 2003; Yu and Buckler, 2006) and marker system (Stich et al., 2006). The analysis of LD patterns among the cauliflower diversity set showed that global LD is moderate as indicated by an average of *r^2^* = 0.06, while 20.71% of all marker pairs showing significant LD estimates (*P* < 0.05). In other crops, for instance, global LD has been reported with an average of *r^2^* = 0.019 to 0.029 in wheat populations (Neumann et al., 2011; Laidò et al., 2014; self-pollinating) or *r^2^* = 0.027 in rapeseed (Ecke et al., 2010; predominantly self-pollinating) and *r^2^* = 0.07 in maize (cross-pollinating; Hoyle et al., 2007; Abrol, 2012). Cauliflower is predominantly cross-pollinating, while expressing variability in self-incompatability (Hadj-Arab et al., 2010). The observed average LD extent in cauliflower is comparable to the values of maize. As it holds true for maize, nearly all commercial cauliflower cultivars are hybrids and parental lines are strongly homozygous inbreds or doubled-haploids (DHs), which may result in the slightly higher global LD compared to self-pollinating species like wheat. Generally, higher LDs are assumed for self-pollinating species than for cross-pollinating species. However, since LD is a population-based phenomenon, the higher LD extent within the cauliflower population might result from reduced genetic diversity due to the population bottleneck in breeding populations compared to those in landraces or crop wild relatives and in particular might also be based on the potential effect of assortative mating based on flowering time, since crosses are made between plants of similar flowering time, while the variation in flowering time within cauliflower is very large. Lines with long and very long maturing time (100–280 DAS) were, for instance, excluded in this study. In addition, differences in population size might also lead to variation in LD extent, since small population sizes often result in an overestimation of background LD (Bouchet et al., 2012). Comparison of mean *r^2^* of linked and unlinked markers with significant LD estimates (*P* < 0.05) with average values of *r^2^* = 0.23 and *r^2^* = 0.06, clearly shows that the major cause for LD in the cauliflower genome is intrachromosomal linkage. Estimation of LD decay, i.e., the rate of return to random association between two given alleles, revealed LD decay in the cauliflower population is relatively slow for cross-pollinating cultivars but comparable to crops with a similar breeding system. Studies in maize reported LD decay ranges from less than 1 kb (Tenaillon et al., 2001) in landraces to >100 kb in elite (more closely related) breeding lines (Ching et al., 2002). Since LD decay defines resolution power of association mapping, cauliflower population has to be mapped with an average of one informative marker per 400 kb to ensure sufficient marker density for performing unbiased GWAS. In cases of low LD extent, a candidate gene approach might be preferred in order to perform association studies because otherwise marker quantity has to be extensively increased to cover the variation in the entire genome.

Gene expression analysis of flowering time genes *BoFLC2* and *BoVRN2* was carried out to evaluate their functional relevance in temperature-regulated curd induction in cauliflower. Results showed increased transcript level of both genes until the onset of curd induction in the medium accession (reliable parent) as well as in the early accession, both exhibiting short time to curding. In contrast, in medium-long accession (unreliable parent), transcript levels of *BoFLC2* and *BoVRN2* were generally lower. Thus, results indicate a positive relation between *BoVRN2* expression and reliability in curd induction under high ambient temperature. That could be attributed to higher capacity of suppressing floral inhibitors like *BoFLC2*; although *BoFLC2* expression levels were generally high. *FLC* is the major repressor of flowering by inhibiting transcription of downstream floral integrator genes, such as *FT* and *AP* (Schmitz and Amasino, 2007). In contrast, expression of *VRN2* accelerates flowering by inhibiting *FLC* und thus releases meristem identity genes from *FLC* repression (Alexandre and Henning, 2008; Deng et al., 2011). In the present study, no consistent reduction in *BoFLC2* transcript level over time could be observed, which gives evidence that different alleles of the various *BoFLC* paralogs may exert different effects on flowering time and/or *BoFLC*-independent signaling pathways regulating floral transition might exist. In *B. oleracea* four different homologs of *BoFLC* had been reported (Okazaki et al., 2007). Recently, Ridge et al. (2014) identified a functional allele of *BoFLC2*, along with a mutated *boflc2* allele (predicted with a loss-of-function) that largely accounts for flowering time variation in cauliflower. *BoFLC2* transcription was reduced with vernalization, inversely with *BoFT*-expression, which is a floral integrator gene. However, no significant contribution of other homologs than *BoFLC2* to flowering time could be proofed yet. Hypothesis that other genes than *BoFLC* might be responsible for flowering time variation in cauliflower could be strengthened by previous QTL study on flowering time variability in a *B. oleracea* DH population primarily based on differences in vernalization response (Uptmoor et al., 2012), although, no co-segregation of *BoFLC* locus with flowering time could be observed. Irwin et al. (2012) suggested other candidate genes like a FRIGIDA ortholog to be involved in temperature-driven floral transition. These findings suggest that *FLC*-independent vernalization pathways, like already reported in *Arabidopsis* (Caicedo et al., 2004; Alexandre and Henning, 2008) might exist and might give explanation that in the present study no consistent *BoFLC*-transcription patterns, generally correlating with variation in curd induction among different genotypes, could be detected.

## Conclusion

The present study indicates high phenotypic variation in temperature-related curd induction among the cauliflower diversity set. GWA mapping revealed significant loci associated with floral transition with respect to different temperature regimes, which possesses basis for selection of genomic loci useful for marker-assisted breeding. Thereby, the investigation of global and local LD patterns defined resolution power of association mapping. Promising QTL regions and candidate genes putatively being involved in vernalization and floral transition were identified and further candidate gene approaches can help to dissect allelic diversity and thus, to elucidate genetic variation in temperature response and floral transition in cauliflower. Understanding of vernalization-induced flowering would potentially accelerate predicting and managing harvest traits in cauliflower production and enforce breeders to integrate marker-assisted strategies that benefit the development of elite cauliflower cultivars adapted to a wide geographical range of cultivation.

## Conflict of Interest Statement

The Associate Editor Thomas Debener declares that, despite being affiliated with the same institute as the authors Yaser Hasan and Ralf Uptmoor, the review process was handled objectively. The authors declare that the research was conducted in the absence of any commercial or financial relationships that could be construed as a potential conflict of interest.

## Footnotes

1 http://www.r-project.org

2 www.ocri-genomics.org/bolbase/

3 https://www.arabidopsis.org/

4 www.nanodrop.com/

5 www.ncbi.nlm.nih.gov/tools/primer-blast

6 www.ncbi.nlm.nih.gov/sra
