# Supplementary material for: Genetic variation of temperature-regulated curd induction in cauliflower: elucidation of floral transition by genome-wide association mapping and gene expression analysis
Source: Front Plant Sci. 2015 Sep 10;6:720. doi: 10.3389/fpls.2015.00720 (PMC4564693; doi:10.3389/fpls.2015.00720)
Supplement: Supplementary file 1 [file Data_Sheet_1.DOCX]

***Supplementary Material***

**Genetic variation of temperature-regulated curd induction in cauliflower: elucidation of floral transition by genome-wide association mapping and gene expression analysis**

**Claudia Matschegewski^1*^, Holger Zetzsche^2^, Yaser Hasan^3^, Lena Leibeguth^1^, William Briggs^4^, Frank Ordon^2^, Ralf Uptmoor^1,3^**

^1^Chair of Agronomy, Faculty of Agriculture and Environmental Science, University of Rostock, Rostock, Germany

^2^Institute of Resistance Research and Stress Tolerance, Julius-Kuehn Institute (JKI), Quedlinburg, Germany

^3^Institute of Horticultural Production Systems, Leibniz Universität Hannover, Hannover, Germany

^4^Syngenta Seeds, B.V., Enkhuizen, The Netherlands

*** Correspondence:** Claudia Matschegewski, Chair of Agronomy, Faculty of Agriculture and Environmental Science, University of Rostock, Justus-von-Liebig-Weg 6, D-18059 Rostock, Germany. claudia.matschegewski@uni-rostock.de

1. **Supplementary Figures and Tables**

## Supplementary Tables

**Supplementary Table 1. Gene specific primer sequences for qRT-PCR.**

| **Gene** | **Primer sequence (forward primer / reverse primer; from 5’ to 3’)** | |
| --- | --- | --- |
| ***BoFLC2*** | TCAAATTAGGGCACAGGGACC | GCTGACGAGCTTTCTCAACG |
| ***BoVRN2*** | TCGTCACAAGAGGAGGAGGT | AAAGGGAGCGAATGCGAAGA |
| ***18S*** | CGAGACCTCAGCCTGCTAACTAG | TCAAACTTCCTTGGCCTAAACG |

**Supplementary Table 2. Genomic location, *P*-level of significance and allele effects for markers significantly associated with curd induction under the temperature regimes GH-T1 and GH-T2.**

| **Temperature regime** | **Chromosome** | **Position (Mb)** | **Marker ID** | ***P*-value** | **Effect**  **rare allele** | **Effect**  **common allele** |
| --- | --- | --- | --- | --- | --- | --- |
| **GH-T1** | **1** | 30.35 | Syn06449 | 0.0012 | -0.1253 | -0.1279 |
|  |  | 30.35 | Syn10351 | 0.0012 | -0.1253 | -0.1279 |
|  |  | 30.35 | Syn11342 | 0.0012 | -0.1253 | -0.1279 |
|  |  | 30.35 | Syn10774 | 0.0012 | -0.1253 | -0.1279 |
|  |  | 30.35 | Syn03920 | 0.0012 | -0.1253 | -0.1279 |
|  | **2** | 26.37 | Syn10742 | 0.0067 | 0.0858 | 0.0466 |
|  |  | 26.38 | Syn13412 | 0.0047 | 0.1493 | 0.1097 |
|  | **3** | 1.96 | Syn08076 | 0.0051 | 0.0000 | -0.0093 |
|  |  | 54.11 | Syn06502 | 0.0042 | 0.0000 | 0.0073 |
|  | **4** | 14.65 | Syn02495 | 0.0058 | 0.0884 | 0.0285 |
|  |  | 14.66 | Syn04530 | 0.0058 | 0.0884 | 0.0285 |
|  |  | 14.66 | Syn08731 | 0.0072 | 0.0934 | 0.0316 |
|  |  | 14.76 | Syn02642 | 0.0058 | 0.0884 | 0.0285 |
|  |  | 14.76 | Syn02700 | 0.0058 | 0.0884 | 0.0285 |
|  |  | 14.76 | Syn02061 | 0.0058 | 0.0884 | 0.0285 |
|  |  | 14.76 | Syn00672 | 0.0058 | 0.0884 | 0.0285 |
|  |  | 14.77 | Syn01924 | 0.0060 | 0.0919 | 0.0241 |
|  |  | 14.80 | Syn06425 | 0.0072 | 0.0934 | 0.0316 |
|  |  | 14.85 | Syn00907 | 0.0058 | 0.0884 | 0.0285 |
|  |  | 14.85 | Syn00725 | 0.0058 | 0.0884 | 0.0285 |
|  |  | 34.49 | Syn00454 | 0.0073 | 0.0156 | 0.0816 |
|  |  | 34.49 | Syn02454 | 0.0073 | 0.0156 | 0.0816 |
|  |  | 34.49 | Syn00944 | 0.0073 | 0.0156 | 0.0816 |
|  |  | 34.49 | Syn05674 | 0.0073 | 0.0156 | 0.0816 |
|  |  | 34.50 | Syn12538 | 0.0073 | -0.0549 | 0.0867 |
|  |  | 34.50 | Syn02745 | 0.0081 | -0.0618 | 0.1282 |
|  | **6** | 15.63 | Syn07211 | 0.0033 | 0.0000 | 0.0431 |
|  |  | 15.67 | Syn07714 | 0.0033 | 0.0000 | 0.0431 |
|  |  | 22.55 | Syn14298 | 0.0018 | 0.0000 | -0.0775 |
|  | **8** | 3.14 | Syn08393 | 0.0043 | 0.1258 | 0.0555 |
|  |  | 6.78 | Syn02332 | 0.0077 | 0.0000 | -0.0207 |
|  |  | 10.88 | Syn05633 | 0.0051 | 0.0592 | -0.0412 |
|  |  | 11.10 | Syn05154 | 0.0012 | 0.0439 | -0.0999 |
|  | **9** | 10.06 | Syn10041 | 0.0070 | -0.0056 | 0.0930 |
|  |  | 10.06 | Syn14118 | 0.0078 | -0.0049 | 0.0902 |
|  |  | 10.06 | Syn02253 | 0.0066 | 0.0021 | 0.1011 |
|  |  | 10.07 | Syn04464 | 0.0068 | 0.0339 | 0.0839 |
| **GH-T2** | **3** | 6.66 | Syn07509 | 0.0025 | 0.0000 | -0.1288 |
|  | **8** | 5.27 | Syn05651 | 0.0089 | 0.1861 | 0.2247 |
|  |  | 5.27 | Syn05942 | 0.0089 | 0.1864 | 0.2247 |
|  |  | 5.28 | Syn01236 | 0.0044 | 0.1502 | 0.1915 |
|  |  | 5.28 | Syn12160 | 0.0041 | 0.0000 | 0.1927 |
|  |  | 5.28 | Syn04278 | 0.0081 | 0.1835 | 0.2258 |
|  |  | 5.62 | Syn06932 | 0.0089 | 0.1466 | 0.1950 |

## Supplementary Figures


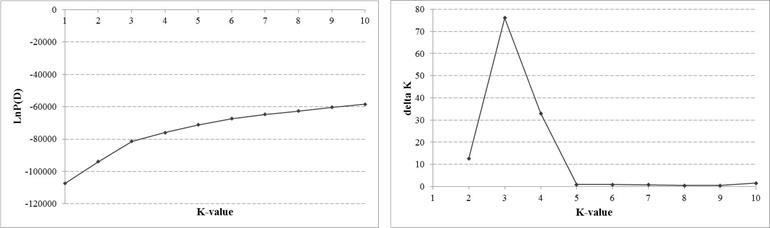


Supplementary Figure 1. Estimation of population structure within the cauliflower diversity set. Population structure was inferred for *K* subgroups subdividing the cauliflower population based on calculation of LnP(D) and Δ*K* according to Evanno et al. (2005). Best *K* was estimated for *K* = 3 seen as LnP(D) plateau (*left*) and a Δ*K* peak at *K* = 3 (*right*).
